# White matter hyperintensities drive propagating grey matter atrophy in cerebral small vessel disease

**DOI:** 10.1093/braincomms/fcaf429

**Published:** 2025-10-30

**Authors:** Ronghua Mu, Peng Yang, Xiaoyan Qin, Wei Zheng, Jian Lv, Bingqin Huang, Xin Li, Yuling Feng, Danyi Huang, Peijia Li, Siyu Dai, Luqi Cheng, Xiqi Zhu

**Affiliations:** Department of Radiology, Nanxishan Hospital of Guangxi Zhuang Autonomous Region, Guilin 541004, China; Department of Radiology, Nanxishan Hospital of Guangxi Zhuang Autonomous Region, Guilin 541004, China; Department of Radiology, Nanxishan Hospital of Guangxi Zhuang Autonomous Region, Guilin 541004, China; Department of Radiology, Nanxishan Hospital of Guangxi Zhuang Autonomous Region, Guilin 541004, China; Department of Radiology, Nanxishan Hospital of Guangxi Zhuang Autonomous Region, Guilin 541004, China; Department of Radiology, Nanxishan Hospital of Guangxi Zhuang Autonomous Region, Guilin 541004, China; Graduate School, Guilin Medical University, Guilin 541002, China; Department of Radiology, Nanxishan Hospital of Guangxi Zhuang Autonomous Region, Guilin 541004, China; Department of Radiology, Nanxishan Hospital of Guangxi Zhuang Autonomous Region, Guilin 541004, China; Department of Radiology, Nanxishan Hospital of Guangxi Zhuang Autonomous Region, Guilin 541004, China; Department of Radiology, Nanxishan Hospital of Guangxi Zhuang Autonomous Region, Guilin 541004, China; Department of Radiology, Nanxishan Hospital of Guangxi Zhuang Autonomous Region, Guilin 541004, China; Graduate School, Guilin Medical University, Guilin 541002, China; School of Life and Environmental Sciences, Guilin University of Electronic Technology, Guilin 541004, China; Department of Radiology, Affiliated Hospital of Youjiang Medical University for Nationalities, Baise 533000, China; Life Science and Clinical Medicine Research Center, The Affiliated Hospital of Youjiang Medical University for Nationalities, Baise 533000, China

**Keywords:** causal structural covariance network, grey matter volume, white matter hyperintensities, magnetic resonance imaging, insula

## Abstract

WMH, a neuroimaging marker of cerebral small vessel disease, is closely associated with cognitive decline and structural brain changes. However, the precise mechanisms through which WMH-associated GMV changes ultimately translate to cognitive decline remain unclear, particularly regarding propagation patterns and causal interactions within affected neural circuits. To investigate the progressive structural changes in WMH patients based on disease severity, we recruited 185 patients with cerebral small vessel disease and 40 healthy controls, who underwent magnetic resonance imaging scans. First, voxel-based morphometry analysis was performed to compare GMV differences between WMH patients and healthy controls, followed by subgroup analyses across different disease stages to identify key regions with significant morphological changes. Subsequently, causal structural covariance network analysis, modularity analysis and functional decoding were employed to map the causal relationships of GMV changes, the hierarchical topography and functional characteristics of the structural network throughout the WMH progression. Finally, mediation analysis was conducted to explore the relationships between WMH volume, GMV, and cognition, providing insights into the underlying causal pathways. The results revealed that GMV reductions originated in the right insula and progressively extended to cortical and subcortical regions with increasing disease severity. Causal structural covariance network analysis identified the right insula as a central hub, exerting causal effects on GMV reductions in regions associated with executive function and attention. Modularity analysis and functional decoding further highlighted key pathways linking the right insula to cortico-subcortical networks involved in cognitive regulation and motor coordination. Additionally, compensatory GMV increases were observed in specific regions, suggesting neuroplastic responses to WMH-related damage. Mediation analysis demonstrated that GMV reductions significantly mediated the relationship between WMH volume and cognitive impairments, particularly in executive function and processing speed. Overall, the right insula acts as a critical hub driving hierarchical GMV atrophy and network disruption in WMH. Its early involvement and causal influence highlight its importance as a potential target for interventions to mitigate cognitive decline.

## Teaser

Mu *et al.* report that white matter hyperintensities trigger grey matter atrophy originating in the right insula. This degeneration propagates through cortico-subcortical structural networks, mediating declines in executive function and processing speed. The insula emerges as a key hub and a potential target for interventions to mitigate cognitive decline.

## Graphical Abstract

**Graphical Abstract:**
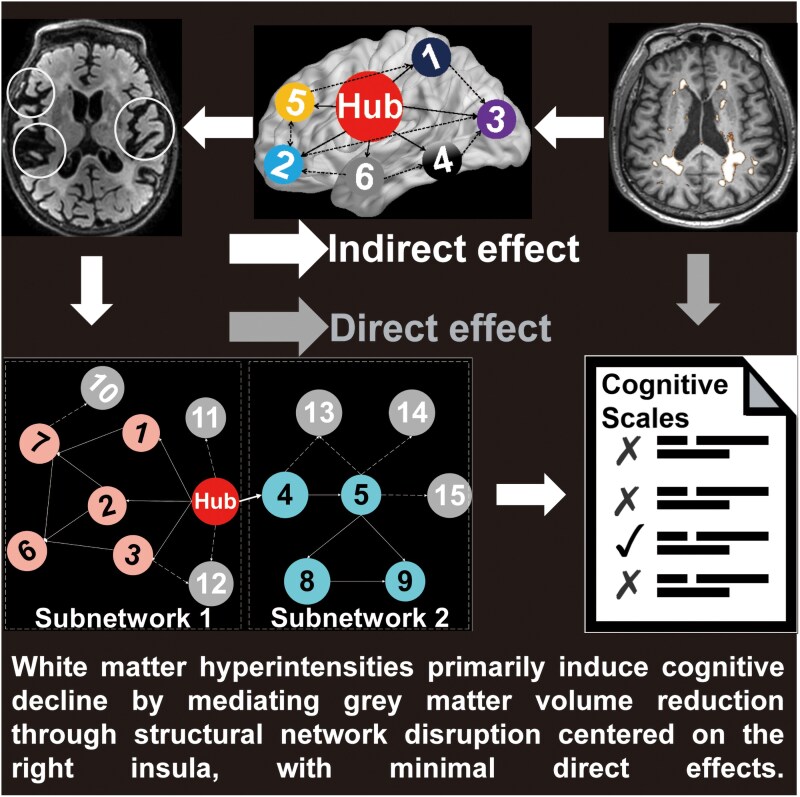

## Introduction

White matter hyperintensities (WMH), a neuroimaging marker of cerebral small vessel disease (CSVD), are linked to impaired cerebrovascular reactivity and progressive grey matter volume (GMV) loss, accelerating cognitive decline.^1^ Although cognitive decline is typically considered uncommon in the early stages of WMH,^2,3^ emerging evidence suggests early pathological effects may impair cognitive function before clinical symptoms appear.^4,5^ While WMH research spans adult populations, mechanistic understanding of mild WMH, particularly during midlife's critical neurodevelopmental window, remains less characterized compared to severe WMH in older cohorts.^6,7^ This gap may underestimate the impact of mild WMH on brain structure and function during midlife, warranting further investigation into early-stage GMV changes.

Previous studies confirm that WMH volume is associated with structural atrophy in cognition-critical regions, including the frontoparietal cortex, insula and caudate nuclei, suggesting secondary neurodegeneration due to white matter damage.^8,9^ While these findings highlight widespread grey matter loss, they offer limited insight into interregional relationships—failing to explain how network-level disconnection accelerates domain-specific cognitive decline. Disrupted white matter pathways may propagate abnormal information flow, leading to distinct atrophy patterns reflective of CSVD pathology.^10^ Previous studies have demonstrated that dysfunction in the salience network and executive control network contributes to the progression of CSVD.^11-13^ However, causal relationships between WMH-impaired regions and their impact on brain network topology remain unknown. This gap necessitates novel approaches to model directional influences across diseased networks.

To address the unresolved causal relationships in network topology, we employed causal structural covariance networks (CaSCN). This framework integrates Granger causality (GC) analysis with structural covariance techniques to model directional pathways of structural change between brain regions.^14^ While GC analysis was originally applied to functional time-series data, its adaptation to morphometric data sequenced by progression information (e.g. disease duration) has successfully characterized structural progression in Parkinson’s disease and schizophrenia.^15,16^

Having established directional atrophy trajectories via CaSCN, we next dissect their damage pathways, behavioural correlates and grey matter mediation effects through integrated multimodal analyses. Building upon CaSCN-mapped directional atrophy pathways, modularity analysis identifies critical damage pathways within structural networks, revealing key routes of neurodegeneration propagation.^17^ Functional decoding maps these pathways onto canonical neurocognitive systems to validate functional coherence in atrophy progression.^18^ And mediation analysis quantitatively delineated the pathway-specific contributions of both the direct effects of WMH and the indirect effects mediated by GMV atrophy on cognitive decline, thereby elucidating the pathways linking network-level structural damage to clinical functional impairment. Together, this approach rigorously maps WMH-driven atrophy progression while elucidating network-mediated cognitive decline mechanisms, bridging localized WM lesions to global dysfunction.

To investigate grey matter changes and their causal pathways in WMH progression, we employed a multi-stage analytical framework. First, voxel-based morphometry (VBM) was applied to identify GMV alterations associated with global WMH volume in patients compared to healthy controls (HCs). Subgroup analyses, based on both WMH volume and Fazekas scale grades, were subsequently conducted to delineate severity-dependent morphological patterns across complementary dimensions of WMH severity. Next, leveraging CaSCN, we constructed directional models of GMV alterations by sequencing morphometric data according to WMH volume—establishing pseudo-temporal trajectories to map structural change propagation. To delineate system-level impacts, network modularity analysis decoded WMH-related damage pathways, with functional characterization of implicated regions conducted via the BrainMap database. Finally, mediation analysis examined whether WMH mediates GM changes to contribute to cognitive decline, thus elucidating the pathways linking WMH, GM alterations and cognitive impairment.

## Materials and methods

### Participants

The study was approved by the Ethics Committee of [Blinded Institution] (2020NXSYEC-006), and informed consent was obtained from all participants. Between May 2020 and November 2021, a total of 329 right-handed participants were recruited, including CSVD patients with WMH and cognitively normal controls. Neurological and cognitive assessments were conducted according to the VICCCS-2 guidelines,^19^ which involved medical interviews and standardized cognitive tests administered by experienced neurologists.

Participants aged ≥40 years were eligible. For patients with WMH, the presence of MRI confirmed WMH (Fazekas grades 1–3) served as a mandatory inclusion criterion, with or without permitted comorbid imaging markers including enlarged perivascular spaces (PVS burden <10 in the hemisphere with higher load across basal ganglia and centrum semiovale regions) or total cerebral microbleeds <2, whereas lacunar infarcts were excluded. These thresholds were chosen to ensure that WMH remained the dominant imaging marker while minimizing the independent contribution of other CSVD lesions to grey-matter atrophy and cognitive impairment. Normal controls, on the other hand, were required to have no neuroimaging markers of CSVD. All participants, including both patients and controls, were required to be dementia-free, meet cognitive thresholds [Montreal Cognitive Assessment (MoCA) ≥26 and Mini-Mental State Examination (MMSE) ≥27, or ≥20 for primary-level education] and independently complete all study procedures.

Exclusion criteria included conditions associated with white matter lesions, such as Alzheimer’s disease, multiple sclerosis, Parkinson’s disease, use of certain medications, infections, inflammation and metabolic disorders. Participants were also excluded if they had a history of neuropsychiatric disorders (e.g. schizophrenia, major depressive disorder, anxiety), intellectual disabilities or severe drug or alcohol abuse. Additional exclusions included primary or metastatic brain tumours, prior brain surgeries, and contraindications to MRI (e.g. pacemakers, cochlear implants). Participants with incomplete or low-quality data (e.g. missing neuroimaging sequences or images with significant motion artifacts as identified by visual inspection) were excluded.

Finally, 185 patients with WMH and 40 HCs were included in the current study. Detailed inclusion and exclusion criteria are presented in Supplementary Fig. S1.

### Clinical assessment

All participants underwent neuropsychological testing across multiple cognitive domains prior to MRI. Assessments included the Beijing version of the MoCA and the MMSE, which evaluate general cognitive function. Cognitive impairment was defined as a MoCA score <26 (with +1 point added to the raw score for participants having ≤12 years of education prior to applying the cutoff) or an MMSE score <27 (≥20 for primary-level education),^20,21^ both of which evaluate general cognitive function. The Digit Symbol Test (DST) and Trail Making Test Part A (TMT-A) were used to assess attention, processing speed, and executive function.^22,23^ For most tests, lower scores indicated greater impairment, except for TMT-A, where longer completion times reflected poorer performance.

### Image acquisition

MRI data were acquired using a 3.0T scanner (Ingenia 3.0CX; Philips Healthcare, Netherlands) with a 32-channel head coil. The imaging protocol for CSVD diagnosis included 3D T1-weighted, 3D T2-weighted, 3D fluid-attenuated inversion recovery (FLAIR), susceptibility-weighted imaging and diffusion-weighted imaging sequences. Detailed parameters are provided in Supplementary Table S1.

### WMH lesion segmentation

Lesions were segmented by the lesion growth algorithm as implemented in the Lesion Segmentation Tool toolbox v3.0.0 (LST v3.0.0; https://www.applied-statistics.de/lst.html) for SPM. The algorithm segmented T1 images into grey matter, white matter and cerebrospinal fluid, combining this information with FLAIR intensities to generate lesion belief maps. An initial lesion map was created using a threshold (κ = 0.30) and expanded along hyperintense voxels in the FLAIR image, resulting in a lesion probability map. Given the limitations of automated methods, manual review and correction by an experienced radiologist (***, 5 years of experience) were conducted to address potential misclassifications, focusing on regions like the scalp, cerebellum and brainstem.

### 3D T1-weighted MRI data preprocessing

High-resolution 3D T1-weighted MRI data were preprocessed using CAT12 (https://neuro-jena.github.io/cat), integrated with SPM. The preprocessing pipeline began with artifact correction, format conversion and manual reorientation to align the anterior commissure. Following these initial steps, the images were normalized to Montreal Neurologic Institute (MNI) space and resampled to 1.5 mm³ isotropic voxels. Tissue segmentation then delineated grey matter, white matter and cerebrospinal fluid. Using the segmented grey mattemaps from all participants, we generated a study-specific template was generated via DARTEL's high-dimensional diffeomorphic registration algorithm. Subsequently, nonlinear modulation corrected volume changes during spatial normalization, applying the DARTEL-derived deformation fields to preserve volumetric information in grey matte images. All preprocessed images underwent rigorous quality control using CAT12’s automated report, which evaluates three key metrics: resolution, noise and bias. Images with a weighted average score ≥80 were retained, as this threshold balances sensitivity. Additionally, all data were manually inspected to exclude cases with severe motion artifacts, incomplete brain coverage, or misalignment after normalization.

After quality control, grey matter images were smoothed using an 8 mm full width at half maximum Gaussian kernel. Subsequently, we generated a study-specific grey matter mask by averaging the modulated grey matter probability maps across all participants. The mask was binarized using a threshold of 0.27 (retaining voxels with >27% grey matter probability), which was determined by the SPM Masking Toolbox (http://www0.cs.ucl.ac.uk/staff/g.ridgway/masking/) through a correlation maximization procedure.^24,25^ This data-driven approach identified the threshold (*r* = 0.94 at threshold = 0.27) that best preserves the linear relationship between GMV and tissue probability, effectively excluding low-probability grey matter voxels that exhibit near-zero variance and would violate Gaussian distribution assumptions.

### Voxel-based morphometric analysis: overall GMV alteration patterns in patients with WMH

GMV differences between the WMH group and HCs were assessed using two-sample *t*-tests. The analysis was conducted with covariates including age, gender, education and total intracranial volume (TIV) to control for potential confounding effects. Statistical significance was determined at *P* < 0.05 with cluster-level false discovery rate (FDR) correction.

### Voxel-based morphometric analysis: stage-specific atrophy patterns in patients with WMH

To analyse progressive GMV alterations in WMH patients, two grouping strategies were used. Local polynomial regression identified three WMH volume thresholds (3.21, 7.78, and 14.96), classifying patients into four stages (Stage I–IV, Fig. S2). A secondary analysis applied the modified Fazekas grading system (grades 1–3 for WMH severity). GMV differences between subgroups and HCs were assessed using two-sample *t*-tests (*P* < 0.05, FDR corrected), controlling for age, gender, education and TIV. Consistent GMV alteration patterns across both grouping strategies confirmed the reliability of the VBM findings and the robustness of the classification approach.

### Causal structural covariance network analysis

A primary goal of this study was to examine whether and how early brain atrophy causally influences other brain networks in patients with WMH. Cross-sectional data were treated as pseudo-time series, ordered by WMH volume for characterizing disease progression, assuming greater grey matter atrophy corresponds to later disease stages. In Stage I of the two grouping strategies, seed-based CaSCNs were constructed using a significant right insula cluster identified in our VBM analysis of WMH stage-related atrophy as the seed region. This cluster (MNI coordinates: 46.5, 15, −6; *P* < 0.05, FDR corrected, cluster size > 100 voxels) was selected based on its significant early atrophy. The choice of the seed coordinate was specifically informed by the grouping method based on ‘Local polynomial regression identified three WMH volume thresholds’. The averaged GMV of the right insula was extracted from the sequenced data to generate a pseudo-time series. Signed-path coefficient GC analysis was performed voxel-wise on GMV-reduced data using the REST toolbox (http://www.restfmri.net). Given the seed’s established role as an early atrophy region, positive GC values (seed to target) indicated that reduced GMV (atrophy) in the seed region Granger-predicted reduced GMV (atrophy) in the target region, suggesting a damaging or driving effect propagating from the seed. Conversely, negative GC values (seed to target) indicated that reduced GMV in the seed region Granger-predicted increased GMV in the target region, suggesting a possible compensatory effect counteracting the seed’s influence. Covariates included age, gender, education, and TIV. Moreover, the GC map was transformed to a *z*-score, and the threshold was set based on cluster-level FDR correction (*P* < 0.05 and cluster size > 200), combined with the conditions of *z* > 1.96 and GC > 0.20.

To further investigate the network topology and directional connectivity among regions of interest (ROIs) obtained from CaSCN analysis, ROI-to-ROI GC analysis was performed. Temporal precedence between regions was assessed using overlapping CaSCN maps. A signed-path coefficient GC analysis was applied to construct a causal network, characterizing the directed influences among ROIs. To keep consistency with the voxel-wise CaSCN analysis, the same threshold was set at GC value > 0.20. Binary and weighted ‘in-degree’ and ‘out-degree’ values were calculated for each ROI. In-degree represents the number or strength of connections entering an ROI, while out-degree reflects connections leaving it. The difference between out-degree and in-degree (‘out-in degree’) was used to quantify the net causal influence of each node and classify ROIs as primary causal sources (higher out-in degree) or causal targets (lower out-in degree) within the network. The primary focus of this ROI-level analysis was on identifying key network hubs (sources versus targets) based on out-in degree; the interpretation of the biological significance (e.g. damaging versus compensatory) of individual GC connections requires integration with the seed-based results and the node’s overall causal role (e.g. high out-in degree nodes are more likely primary drivers where positive out-connections may propagate atrophy).

Notably, our correlation analyses (Fig. S3) revealed that WMH volume showed no significant association with age (*r* = 0.143, *P* > 0.05), while GMV exhibited only a weak age-related decline (*r* = −0.155, *P* < 0.05). Crucially, the strong negative correlation between WMH volume and GMV (*r* = −0.954, *P* < 0.001) suggests that the observed causal relationships are more likely to reflect WMH-specific pathological processes rather than generalized age-driven atrophy.

### Modularity analysis

To analyse the topological characteristics of the ROI-based CaSCN, we examined the directed and weighted network using Newman’s modularity optimization algorithm. This method, widely employed in network science, identifies the most optimal division of a network into distinct modules by maximizing the modularity index. The number of modularity reflects the extent to which a network can be partitioned into groups where nodes (ROIs) exhibit stronger intra-module connections compared to inter-module connections. This analysis allows the identification of cohesive subnetworks that are structurally interconnected.

### Functional decoding

To explore the cognitive and functional roles of the identified modules or pathways, the BrainMap database was utilized to examine behavioural domains linked to specific tasks through forward inference. Taxonomic labels (e.g. domains or subdomains) were assigned by comparing activation probabilities within the identified modules to the average activation probabilities across the database. Labels with significantly higher probabilities of activation were interpreted as representing the functional roles of the respective modules. Statistical significance was assessed using a binomial test (*P* < 0.05) with multiple comparisons corrected using FDR.

### Evaluating the mediating role of GMV in the effects of WMH on cognitive function

We performed mediation analysis to examine both direct and indirect effects of WMH volume on executive function and attention, using global GMV as the mediator. Importantly, the GMV measure represented whole-brain volume quantification rather than regional measures derived from CaSCN. All paths in the model were adjusted for age, gender, education and TIV. This approach aimed to clarify how WMH impacts cognitive performance through changes in GMV integrity. Using Maximum Likelihood Estimation, a path model was constructed to assess the relationships among WMH, GMV and cognitive outcomes. Significance of the indirect effects from WMH to GMV and then to cognition was assessed using bias-corrected bootstrap confidence intervals based on 5000 resamples; effects were deemed significant if the 95% confidence intervals did not include zero. In the model, WMH was specified as the independent variable, GMV as the mediator, and two outcome measures: the DST and 1/TMT-A. The inverse of TMT-A (1/TMT-A) was used to align its interpretation with that of the DST, reflecting its inverse relationship with cognitive performance. The model estimated direct, indirect and total effects, providing a comprehensive analysis of how WMH influences executive function and attention via GMV alterations.

### Statistical analyses

The Shapiro–Wilk test and Bartlett’s test were used to assess the normality and homogeneity of variances, respectively, ensuring the suitability of parametric statistical analyses. Continuous data following a normal distribution are presented as mean ± SD, while categorical data are reported as counts and percentages [*n* (%)]. Baseline characteristic differences between the control group, WMH group, and its subgroups were analysed using *t*-tests (for pairwise comparisons), one-way ANOVA (for multiple-group comparisons) and chi-square tests (for categorical variables). All statistical analyses were performed using R Statistical Software (v4.3.2; R Core Team, 2023). Statistical significance was defined as a two-sided *P* < 0.05.

**Figure 1:**
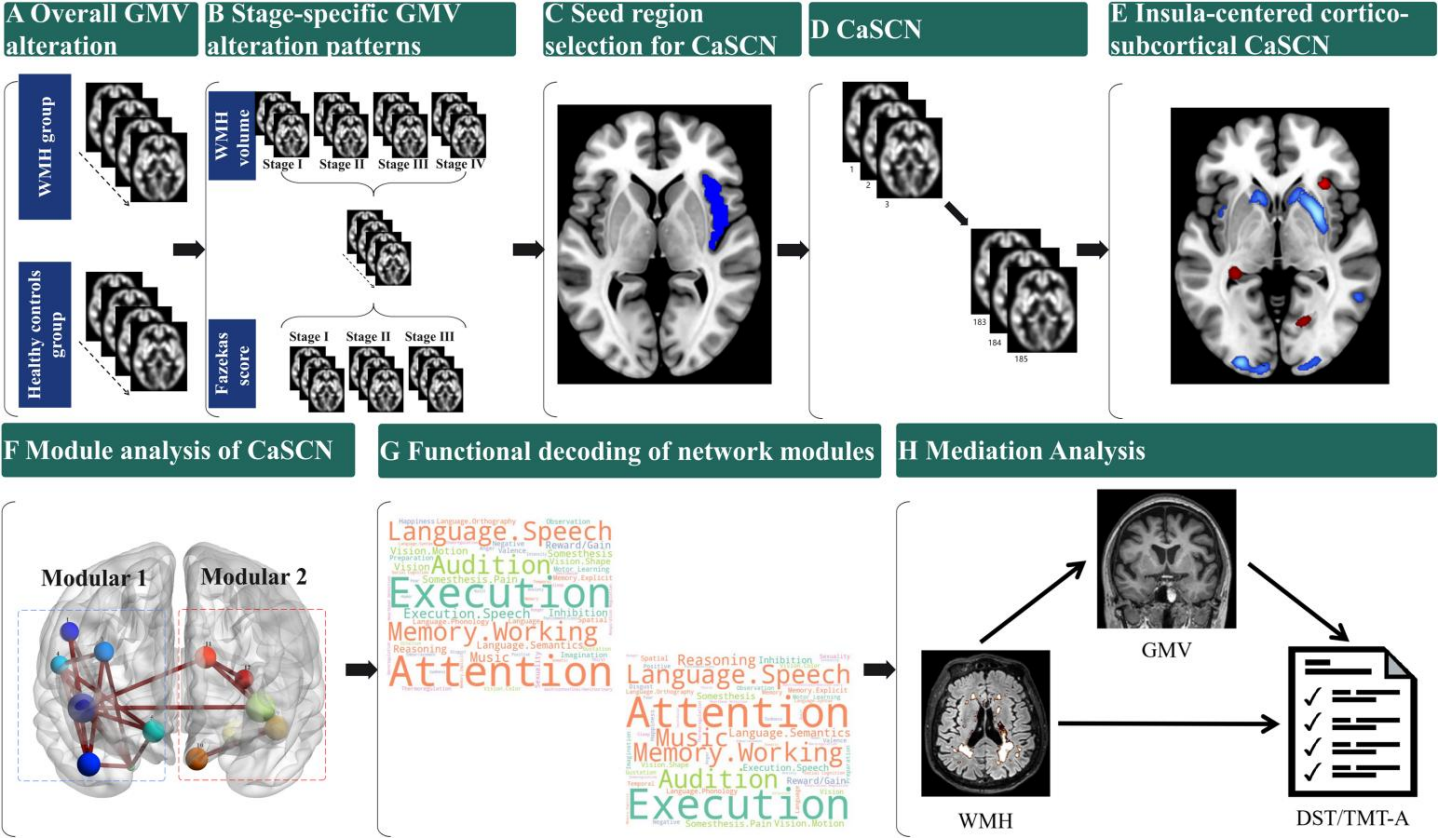
**Processing pipeline.** Pipeline showing a step-by-step process of statistical analysis. (**A**) Overall GMV alterations: Comparison of GMV between WMH patients and HCs using two-sample *t*-tests to identify group-wise differences in GMV. (**B**) Stage-specific GMV atrophy patterns: Two staging strategies were applied to characterize WMH progression: (i) local polynomial regression divided patients into four stages and (ii) modified Fazekas grading system divided patients into three stages. Two-sample *t*-tests were used to compare GMV between each WMH stage and HCs, respectively. (**C**) Seed region selection for CaSCN: Based on stage-specific GMV atrophy patterns, the right insula was selected as the seed region for subsequent CaSCN. (**D**) Construction of CaSCN: GMV images of cerebral small vessel disease patients were sorted by WMH volume, treating WMH volume as a ‘pseudo-time series’ to model progression. (**E**) Insula-centred cortico-subcortical CaSCN: CaSCN analysis was performed using GC analysis to map cortico-subcortical networks seeded from the right insula. Colour bars indicate GC values: cold colours represent positive GC (decreased GMV in one region precedes reduced GMV in another), while warm colours represent negative GC (increased GMV in one region follows reduced GMV in another). (**F**) Module analysis of CaSCN: Newman’s modularity optimization algorithm was used to analyse ROI-based CaSCN topology and identify the optimal module partition of the network. (**G**) Functional decoding of network modules: Using forward inference on the BrainMap database, cognitive function attributes of the identified network modules were analysed to decode their functional characteristics. (**H**) Mediation analysis of WMH, GMV and cognitive function: A mediation model was constructed to examine the direct and indirect effects of WMH on executive function and attention (assessed via DST and TMT Part-A), with GMV as the mediator. Path models were estimated using maximum likelihood.

The processing pipeline is shown in Fig. 1.

## Results

### Demographic and clinical characteristics

**Table 1** summarizes the demographic and clinical characteristics of the study participants. Significant differences were observed between patients with WMH and HCs in key variables, including age, DST scores, TMT-A scores, GMV and TIV. No significant differences were identified in other variables. Supplementary Fig. S3 presents a scatterplot matrix illustrating the relationships between brain volume measures and cognitive performance, highlighting trends across the study population. Additional details on the primary and validation grouping strategies are provided in Supplementary Tables S2 and S3, respectively.

**Table 1:** Demographic and clinical information of WMH and HC groups

Characteristic	WMH (*n* = 185)	HC (*n* = 40)	Statistic	*P*-value
Age (years)	57.7 ± 6.5	45.5 ± 7.8	−10.405	**<0**.**001**^a^
Gender (male/female)	86/99	21/19	0.477	0.490^b^
Educational level (years)	10.8 ± 3.06	11.5 ± 5.4	1.108	0.269^a^
MoCA (0–30)	27.37 ± 1.28	27.50 ± 1.43	0.556	0.579^a^
MMSE (0–30)	27.94 ± 1.08	28.13 ± 1.07	1.009	0.314^a^
DST (correct matches/2 min)	50.63 ± 18.28	41.42 ± 12.33	−3.035	**<0**.**001**^a^
TMT-A (seconds)	41.30 ± 9.91	54.48 ± 15.12	6.868	**<0**.**001**^a^
WMH volume (mm³)	5.24 [2.64, 11.68]	0	NA	NA
GMV (mm³)	548.92 ± 25.81	592.64 ± 46.92	8.200	**<0**.**001**^a^
TIV (mm³)	1424.74 ± 110.79	1505.07 ± 147.42	3.903	**<0**.**001**^a^

Data are presented as median (25th percentile, 75th percentile) for WMH volume and as mean ± standard deviation for all other continuous variables, with gender specified as male/female counts.

Significant differences are highlighted in bold.

^a^Independent Samples *t*-test.

^b^Chi-square *t*-test.

### Voxel-based morphometric analysis of overall GMV alteration in patients with WMH

**Figure 2:**
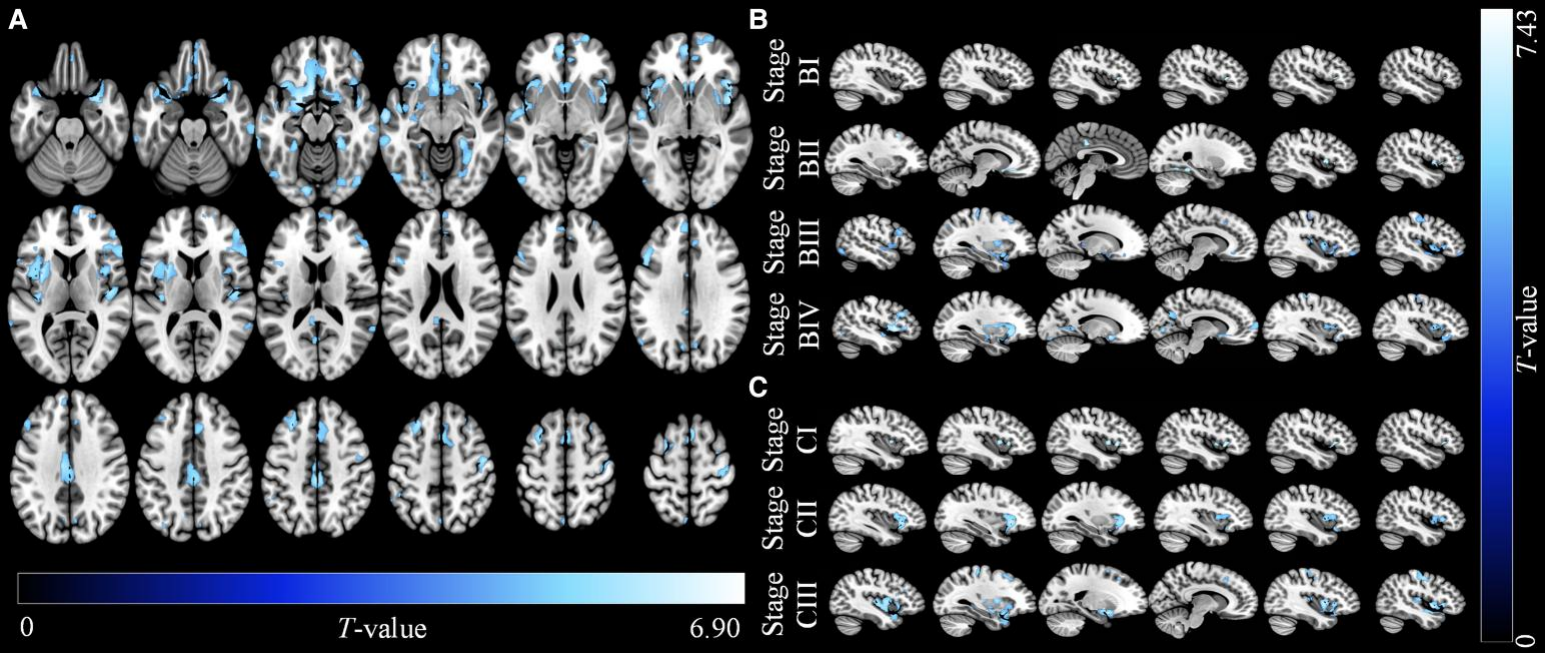
**MR images showing GMV reductions in patients with WMH**. (**A**) Overall GMV reductions in WMH patients compared to HCs. MR images show reduced GMV (blue) in WMH patients (*N* = 185), overlaid on an axial template. Compared to the HCs (*N* = 40), no brain regions with increased volume were found. The colour bar represents *T* values from a two-sample *t* test (*P* < 0.05, FDR correction), with blue indicating regions of significant GMV reduction. Stage-specific GMV reductions in WMH patients relative to the HCs. MR images of reduced GMV were overlaid on a sagittal template. The colour bar represents *T* values from a two-sample *t* test (*P* < 0.05, FDR correction), where the colour scale indicates the degree of GMV reduction. Two staging strategies were applied to characterize WMH progression: (**B**) Subjects with WMH were categorized into four subgroups according to local polynomial regression of WMH volume (stage BI, 0–3.21, *N* = 55; stage BII, 3.21–7.78, *N* = 43; stage BIII, 7.78–14.96, *N* = 50; stage BIV, > 14.96, *N* = 37). (**C**) Subjects with WMH were categorized into three subgroups based on the Fazekas grading grouping strategy (stage CI, Fazekas **I**, *N* = 88; stage CII, Fazekas II, *N* = 52; stage CIII, Fazekas III, *N* = 45).

Compared to HCs, patients with WMH showed significant GMV reductions in multiple brain regions, including the left median cingulate and paracingulate gyri, supplementary motor area, cuneus and angular gyrus; the right precentral gyrus; and the bilateral insula, fusiform gyrus, precuneus and cortical regions across the frontal, parietal, temporal, and occipital lobes. Notably, no significant GMV increases were observed in the WMH group. Detailed results are presented in Fig. 2A and Supplementary Table S4.

### Stage-specific GMV alteration patterns in patients with WMH

The analysis of GMV alterations across WMH stages revealed distinct and progressive patterns of atrophy. In Stage I, GMV reductions were confined to the right insula, indicating early focal atrophy. By Stage II, atrophy expanded to the left gyrus rectus, insula, cingulate and paracingulate gyri, middle frontal gyrus and the right fusiform and middle frontal gyri. In Stages III and IV, further GMV reductions were observed in additional regions, including the left fusiform gyrus, thalamus, supramarginal gyrus, and cingulate and paracingulate gyri; the right parahippocampal gyrus, rolandic operculum and precentral gyrus; as well as bilateral regions such as the precuneus, supplementary motor area, lingual gyrus and widespread cortical areas across the frontal, parietal, temporal and occipital lobes (Fig. 2B).

A similar progression of GMV reductions was observed when WMH stages were defined using the modified Fazekas scale (Fig. 2C). In Stage I, atrophy remained restricted to the right insula. By Stage II, reductions extended to the left middle temporal gyrus, inferior frontal gyrus (triangular part), insula, middle occipital gyrus and cingulate and paracingulate gyri and the right inferior frontal gyrus (orbital part), inferior temporal gyrus, parahippocampal gyrus and cuneus. By Stage III, additional regions were involved, reflecting the cumulative impact of WMH burden on brain structure.

These stage-specific GMV alterations highlight the insula as an early hub of GMV changes, potentially driving progressive brain atrophy and cognitive decline. Detailed results of the stage-specific GMV changes are presented in Supplementary Tables S5 and S6.

### Causal effects of GMV pattern of the right insula

Based on the stage-specific GMV alteration patterns observed in patients with WMH, the right insula, identified as a region of early and significant atrophy in Stage I, was selected as the seed region for the voxel-wise CaSCN. This analysis aimed to reveal distinct causal relationships between the right insula and other brain regions.

**Figure 3:**
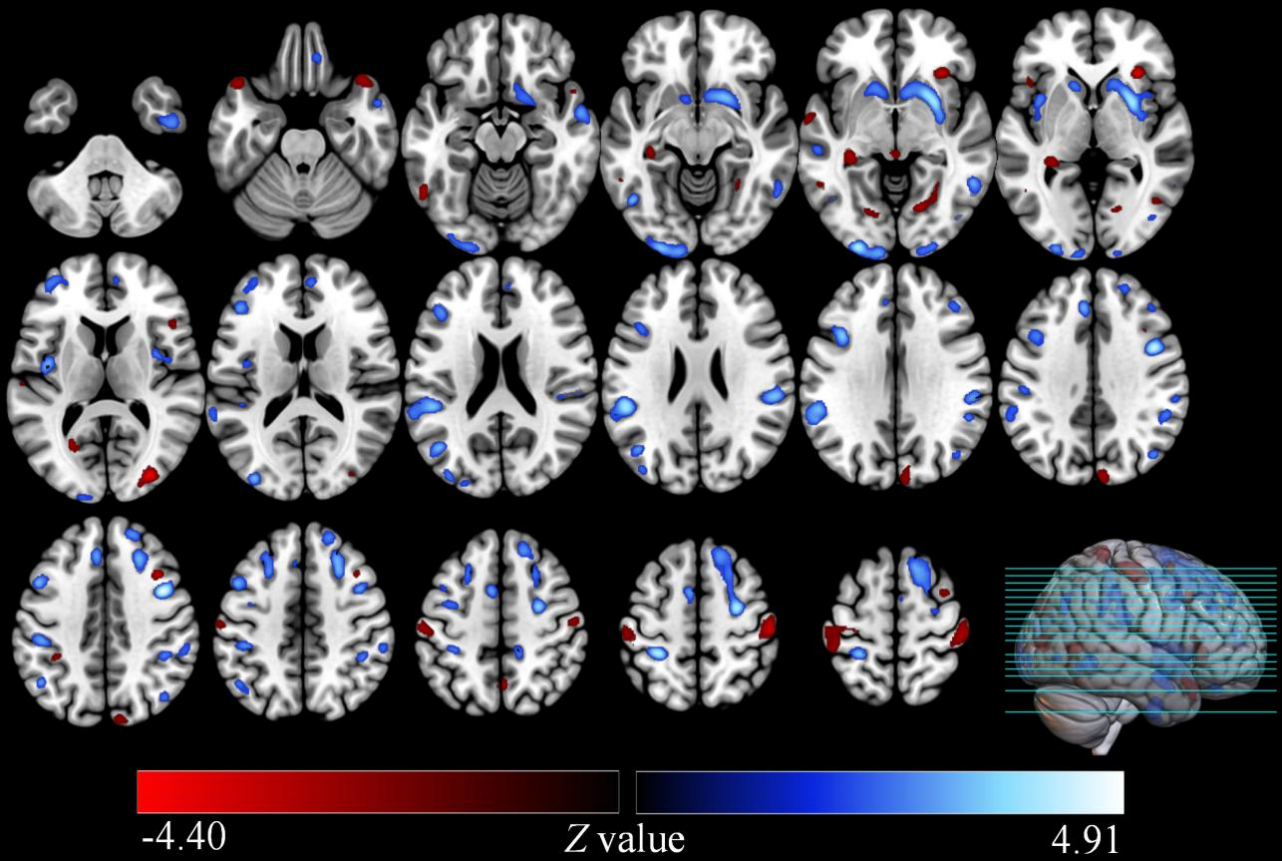
**Causal networks show causal effects of grey matter atrophy pattern in patients with WMH.** Causal networks were constructed by applying CaSCN to sequenced morphometric data, ranked by WMH volume from low to high. The right insula (Montreal Neurological Institute coordinates: 16.5, 15, −6) was used as the seed region based on voxel-based morphometric analysis (*N* = 185). The CaSCN analysis revealed significant causal connections, with the minimum significant GC value being 0.24 (*Z* = 2.58, FDR correction *P* < 0.05). The colour bar represents *Z* values (standardized transformation of GC values). Brain images display multiple axial and sagittal slices, with the rightmost panel showing a 3D rendering of the brain with green lines indicating slice positions for cross-sectional visualization. The cold colour bar represents positive *Z* values (standardized transformation of GC values, with colour intensity corresponding to the strength of GC), indicating that the GMV of these regions decreased following a reduction in the GMV of the right insula. Similarly, the warm colour bar represents negative GC values, suggesting that the GMV of these regions increased after the GMV of the right insula decreased. Statistical thresholds were defined using cluster-level FDR correction (*P* < 0.05) combined with a cluster size > 200 voxels, and voxel-level criteria of |*z*| > 1.96 (corresponding to *P* < 0.05) and GC value > 0.20.

Positive GC values indicate that GMV reductions in the right insula are associated with subsequent reductions in multiple brain regions. On the left hemisphere, GMV reductions were observed in the postcentral gyrus, middle occipital gyrus, inferior frontal gyrus (opercular part), superior frontal gyrus (medial), supplementary motor area, insula, caudate nucleus, and middle frontal gyrus. On the right hemisphere, reductions were found in the lenticular nucleus (putamen), precentral gyrus, and inferior temporal gyrus. Bilaterally, reductions were noted in the supramarginal gyrus and middle temporal gyrus. Conversely, negative GC values suggest potential compensatory mechanisms involving GMV increases. In the left hemisphere, increases were observed in the calcarine fissure, surrounding cortex and hippocampus. On the right hemisphere, increases were found in the middle occipital gyrus, postcentral gyrus, lingual gyrus, temporal pole (including the superior temporal gyrus) and cuneus. These changes may represent adaptive responses aimed at preserving cognitive functions despite structural damage. Detailed results are provided in **Table 2** and Fig. 3.

**Table 2:** Brain regions showing causal effect from the seed of the right insula by using CaSCN analysis

Brain regions	MNI coordinates (*x*,*y*,*z*)			GC value	*Z*-value	Number of voxels
Positive causal effect from the seed of the right insula						
Lenticular nucleus, putamen, right	37.5	−1.5	4.5	3.4255	4.8551	1936
Precental gyrus, right	27	−6	52.5	2.9512	4.4774	3030
Postcentral gyrus, left	−25.5	−39	57	2.7402	4.2998	580
Middle occipital gyrus, left	−25.5	−96	−4.5	2.7392	4.2989	1015
Supramarginal gyrus, left	−49.5	−36	24	2.5966	4.175	1194
Inferior frontal gyrus, opercular part, left	−36	12	31.5	2.0119	3.6275	943
Supramarginal gyrus, right	52.5	−30	27	1.9858	3.6012	897
Middle temporal gyrus, left	−42	−66	22.5	1.9106	3.5249	258
Middle temporal gyrus, right	55.5	0	−19.5	1.8824	3.4959	403
Superior frontal gyrus, medial, left	−6	31.5	36	1.8495	3.4617	289
Supplementary motor area, left	−6	4.5	52.5	1.7905	3.3998	203
Insula, left	−40.5	−7.5	7.5	1.7549	3.3619	414
Inferior temporal gyrus, right	51	−6	34.5	1.4689	3.0427	365
Caudate nucleus, left	−12	15	−3	1.3875	2.9464	348
Middle frontal gyrus, left	−37.5	49.5	9	1.2419	2.7669	352
Negative causal effect from the seed of the right insula						
Middle occipital gyrus, right	30	−84	7.5	1.8596	−4.3566	289
Postcentral gyrus, right	51	−21	55.5	1.0524	−3.4014	650
Calcarine fissure and surrounding cortex, left	−25.5	−60	7.5	1.0375	−3.3807	351
Lingual gyrus, right	18	−67.5	−3	0.73561	−2.9281	207
Hippocampus, left	−30	−34.5	−3	0.67394	−2.825	258
Temporal pole, superior temporal gyrus, right	42	21	−25.5	0.63135	−2.7512	292
Cuneus, right	7.5	−85.5	33	0.5827	−2.6638	251

**Figure 4:**
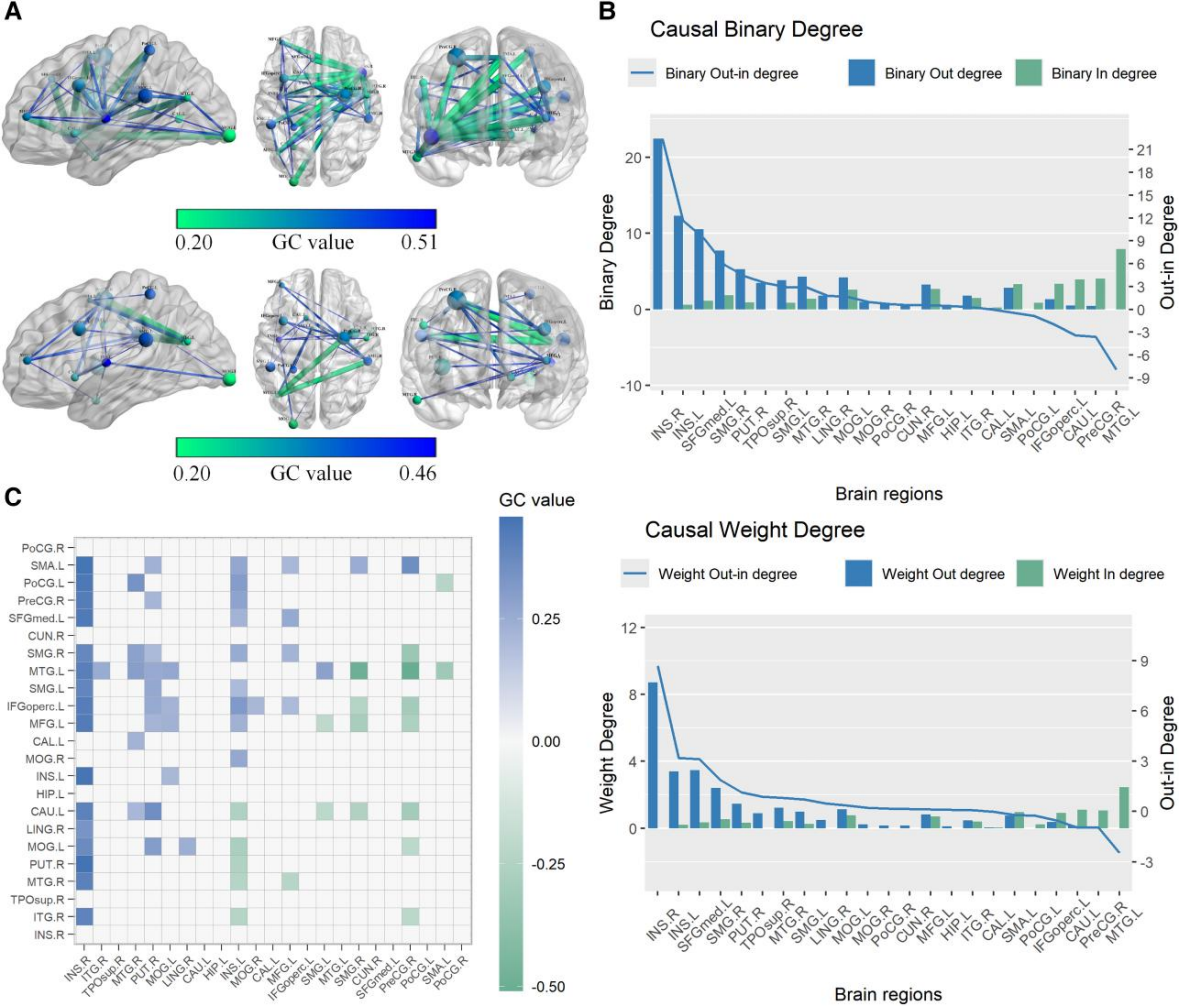
**Causal connectivity among the ROI-wise CaSCN.** All analyses were performed on ROI-based CaSCN (*P* < 0.05, FDR corrected) derived from pseudo-time series data constructed by ranking participants according to WMH volume (*N* = 185). (**A)** The results of a bivariate signed-path coefficient Granger causality analysis performed on a ROI-based causal structural covariance network, illustrating the causal relationships between ROIs. (**B)** The results of a ROI-based CaSCNc, showing the causal relationships between ROIs. The top panel (causal binary degree) shows binary out-degree (cobalt blue bars: sum of directional paths from the ROI to other nodes), binary in-degree (teal bars: sum of directional paths from other nodes to the ROI) and binary out-in degree difference (cobalt blue line: out-degree minus in-degree, indicating net causal source level). The bottom panel (causal weight degree) shows weighted out-degree (cobalt blue bars: sum of path strengths from the ROI to other nodes), weighted in-degree (teal bars: sum of path strengths from other nodes to the ROI) and weighted out-in degree difference (cobalt blue line: weighted out-degree minus in-degree, reflecting net causal influence strength). (**C)** ROI-level CaSCN generated through bidirectional signed-path coefficient Granger causality analysis using an asymmetric matrix, highlighting inter-ROI causal connectivity. ANG, angular gyrus; CAL, calcarine cortex; CAU, caudate; CUN, cuneus; HIP, hippocampus; IFGoperc, inferior frontal gyrus (opercular part); INS, insula; ITG, inferior temporal gyrus; LING, lingual gyrus; MFG, middle frontal gyrus; MOG, middle occipital gyrus; MTG, middle temporal gyrus; PoCG, postcentral gyrus; PreCG, precentral gyrus; PUT, putamen; SMA, supplementary motor area; SMG, supramarginal gyrus; TPOsup, temporal pole (Superior); L, left; R, right.

Figure 4 illustrates the complex causal network among 23 regions of interest. Causal connections originating from the right insula to the left insula, left supplementary motor area, left postcentral gyrus and right putamen exhibited the highest positive GC values. Notably, no negative GC connections were observed originating from the right insula (Fig. 4A and C). The bilateral insula and left superior frontal gyrus (medial) emerged as the regions with the highest out-degree values, projecting causal effects to multiple other regions. In contrast, the left middle temporal gyrus exhibited the highest in-degree, receiving causal inputs from other regions (Fig. 4B).

### Modularity analyses and functional decoding

**Figure 5:**
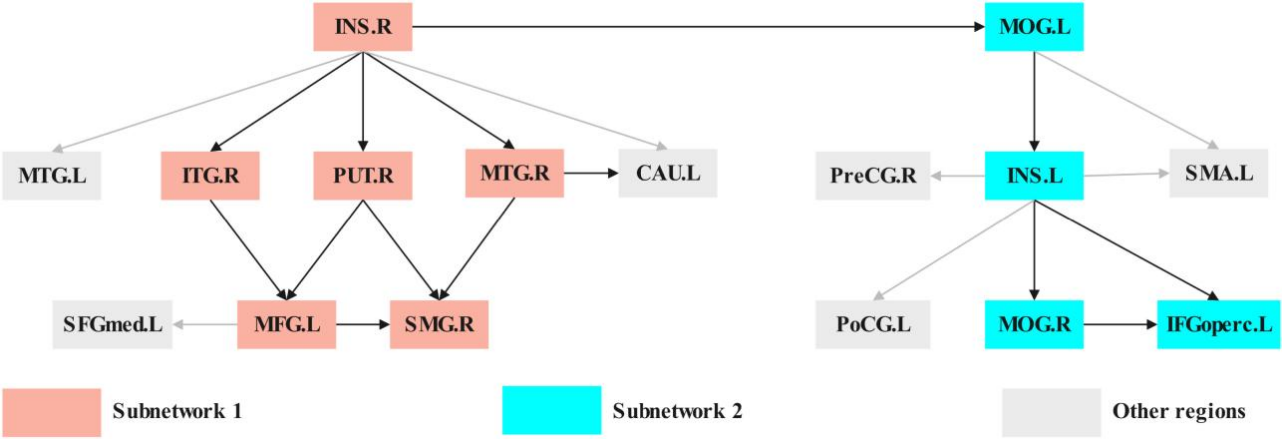
**Modularity analyses.** All analyses were performed in WMH patients (*N* = 185). Modularity analysis using Newman's optimization algorithm identified two distinct subnetworks in WMH patients: Subnetwork 1 (light coral nodes) consisted of the pathways INS.R—ITG.R/PUT.R/MTG.R—MFG.L/SMG.R—SMG.R, and Subnetwork 2 (cyan nodes) included INS.R–MOG.L–INS.L—MOG.R/IFGoperc.L–IFGoperc.L, with grey represented other regions outside these defined subnetworks. Gray nodes represent regions outside these two subnetworks, and arrows indicate directionality of causal relationships between regions of interest. The algorithm maximized modularity to determine the optimal number of modules, resulting in two distinct subnetworks. CAU, caudate; IFGoperc, inferior frontal gyrus (opercular part); INS, insula; ITG, inferior temporal gyrus; MFG, middle frontal gyrus; MOG, middle occipital gyrus; MTG, middle temporal gyrus; PoCG, postcentral gyrus; PreCG, precentral gyrus; PUT, putamen; SFGmed, Superior frontal gyrus (medial); SMA, supplementary motor area; SMG, supramarginal gyrus; **L**, left; R, right.

The modularity analysis of the ROI-based CaSCN identified two distinct subnetworks (Fig. 5). The first subnetwork consisted of the pathways INS.R—ITG.R/PUT.R/MTG.R—MFG.L/SMG.R—SMG.R (right insula—right inferior temporal gyrus/right putamen/right middle temporal gyrus—left middle frontal gyrus/right supramarginal gyrus—right supramarginal gyrus), while the second included MOG.L—INS.L—MOG.R/IFGoperc.L—IFGoperc.L (left middle occipital gyrus—left insula—right middle occipital gyrus/left inferior frontal gyrus—left inferior frontal gyrus). Moreover, the INS.R showed a positive projection to MOG.L, which might suggest that the subnetwork of MOG.L was also driven by INS.R.

To investigate the functional roles of these pathways, a quantitative forward inference analysis was performed to identify the associated behavioural domains. The results revealed that these pathways are primarily involved in executive function and attention (Fig. S4A and B). These findings suggest that the right insula, given its high causal influence in the network (as indicated by centrality metrics) and the functional associations of its pathways with executive function and attention, may act as a potential hub in networks relevant to cognitive processes frequently impaired in WMH-related pathology.

### Evaluation of direct and indirect effects of WMH on executive function and attention

**Figure 6:**
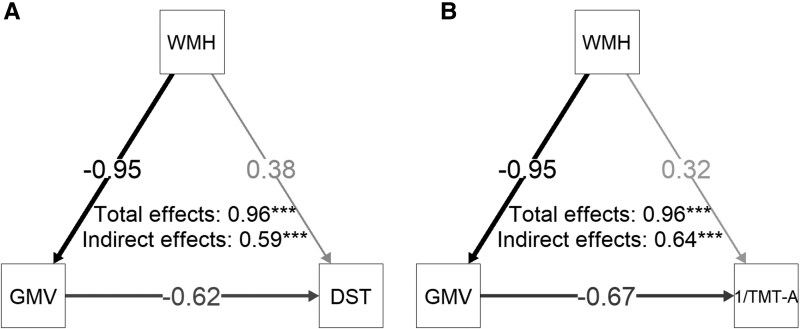
**Path diagrams of direct and indirect effects of WMH on DST and 1/TMT-a.** Path diagrams illustrate the mediating role of GMV in the relationship between WMH and cognitive performance (DST and 1/TMT-A) in WMH patients (*N* = 185), analysed using mediation analysis with maximum likelihood estimation. (**A**) The left diagram shows the effect of WMH on DST, where WMH's direct effect accounted for 39% of the total effect [β = 0.38, 95% CI (0.28, 0.47), *T* = 7.83, *P* < 0.001] and the GMV-mediated indirect effect contributed 61% [β = 0.59, 95% CI (0.49, 0.68), *T* = 12.14, *P* < 0.001], while (**B**) the right diagram depicts the effect of WMH on 1/TMT-A (inverse of TMT-A, aligning with DST's interpretation framework as higher values reflect better cognitive function), with WMH's direct and indirect effects representing 33.4% [β = 0.32, 95% CI (0.24, 0.41), *T* = 7.16, *P* < 0.001] and 66.6% [β = 0.64, 95% CI (0.56, 0.73), *T* = 14.87, *P* < 0.001] of the total effect, respectively. Path line thickness indicates the magnitude of standardized effect sizes, with significant paths (*P* < 0.05) derived from maximum likelihood estimation in mediation models.

To explore the mechanisms linking WMH to cognitive performance, a mediation analysis was conducted to assess the role of GMV as a mediator in this relationship. Mediation analysis revealed that GMV partially mediated the relationship between WMH and cognitive performance DST and 1/TMT-A (Fig. 6 and Supplementary Table S7). Key findings demonstrated: for DST, WMH's direct effect accounted for 39% of the total effect *(P* < 0.001), while the GMV-mediated indirect effect contributed 61% (*P* < 0.001); regarding 1/TMT-A, the direct and indirect effects represented 33.4% (*P* < 0.001) and 66.6% (*P* < 0.001) of the total effect, respectively. These results substantiate the pivotal role of GMV atrophy in WMH-associated cognitive decline.

## Discussion

We identified a progressive stage-specific pattern of brain atrophy and revealed an insula-associated causal degeneration network in patients with CSVD-related WMH. Early selective structural vulnerability was first observed in the right insula, with damage subsequently spreading to widespread cortico-subcortical networks as the disease progressed. By analysing voxel-wise and ROI-wise CaSCNs based on sequenced cross-sectional morphometric maps reflecting WMH progression patterns, we further demonstrated the right insula's causal influence on structural damage in other brain regions through both positive and negative pathways. Notably, this stage-specific vulnerability of the right insula remained evident even after accounting for the influence of age, suggesting that WMH-related pathological processes may drive regional neurodegeneration beyond general ageing effects. Mediation analysis revealed that GMV atrophy serves as a critical mechanism linking WMH burden to cognitive impairments, particularly in processing speed and executive function. These findings highlight the pivotal role of the right insula in WMH pathology and provide novel insights into its function as a causal hub driving hierarchical structural-cognitive decline pathways.

We observed widespread GMV reductions in WMH patients, particularly in the right insula and cortical-subcortical regions, compared to HCs. This aligns with established associations between WMH and CSVD-related structural changes.^12,26^ Crucially, we identified a stage-specific GMV reduction pattern: right insular atrophy emerges early in WMH progression, preceding damage in other regions as WMH burden increases. While bilateral insular atrophy with WMH progression has been documented,^9,27^ our findings specifically implicate the right insula as both an early biomarker and central hub in WMH pathogenesis. It is worth noting that the early vulnerability of the right insula occurred within a relatively narrow age range, reinforcing the notion that WMH-related atrophy may follow distinct patterns that are not solely attributable to normal ageing. Meta-analytic evidence suggests insular GMV loss may reflect remote effects of WMH-induced white matter damage.^28^ Functionally, as a ventral attention network hub, early right insular atrophy may disrupt attentional processing through impaired connectivity with temporoparietal and prefrontal regions.^29,30^ These results establish WMH-related right insular atrophy as a structural substrate for cognitive deficits and a potential monitoring target for early intervention.

Building on the right insula's identification as an early atrophy site in WMH progression, CaSCN analysis revealed its causal influence on widespread GMV reductions. We found right insular atrophy precedes structural damage in connected cortical-subcortical regions, confirming its role as a propagation driver. Positive causal effects suggest neurodegenerative-like spread from primary to downstream areas,^31,32^ potentially mediated by WMH-induced white matter damage through axonal injury and remote effects.^33^ Mechanistically, insular dysfunction may impair its role as an executive control ‘gatekeeper’, disrupting coordination between executive and default mode networks.^34,35^ This aligns with population studies showing WMH-associated GMV reductions correlate with processing speed decline,^36^ cortical thinning in ageing-sensitive regions^8^ and grey–white matter alterations even in mild WMH.^37^ Our findings establish the right insula's dual role as both early biomarker and structural damage propagator in WMH progression.

Negative causal effects indicate right insular GMV reductions may drive compensatory volume increases in connected regions, reflecting neuroplasticity-mediated structural reorganization against WMH damage. Potential mechanisms include synaptogenesis and angiogenesis enhancing functional connectivity and vascular remodeling.^38^ Supporting evidence shows WMH-related cortical thickening in specific areas^39,40^ and preserved cognitive functions through compensatory network recruitment.^41,42^ Our findings suggest this insula-driven damage–adaptation balance may temporarily maintain cognitive resilience, though long-term decompensation risks remain.

Furthermore, our modularity analyses identified two neural pathways originating from the right insula, further supporting its role as an early and critically impaired pathological region in WMH. The first (right insula—temporal lobe—putamen—middle frontal gyrus—supramarginal gyrus) disrupts motor learning and executive-attentional networks through putamen atrophy (ischaemic/demyelination injury),^43,44^ temporal lobe degeneration (memory/executive deficits)^36,45^ and supramarginal gyrus damage (attentional disorientation).^46,47^ In contrast, the second pathway (right insula—bilateral middle occipital/left inferior frontal gyri) impairs visual-cognitive integration via occipital cortex atrophy (spatial attention deficits)^48,49^ and frontal dysfunction (working memory-task integration).^50,51^ Functional mapping demonstrated both pathways’ strong associations with attentional-executive network dysfunction. These findings collectively illustrate how right insula-mediated GMV reductions propagate WMH-related structural damage through specialized yet interconnected circuits, solidifying its role as a pivotal hub for structural–functional integration in the brain.

Our mediation analysis revealed that while WMH volume directly impacts cognitive performance, its effects are predominantly mediated by GMV alterations. WMH drives cognitive decline through structural mechanisms including cortical thinning, white matter tract damage and disrupted functional connectivity, with GMV reductions acting as the central mediator.^50^ This aligns with CSVD studies showing GMV mediates WMH-cognition relationships,^9,31^ particularly in frontal/posterior regions critical for psychomotor speed and executive function.^26,52,53^ Although age is a well-established contributor to both WMH accumulation and GMV reduction, our findings suggest that in this cohort, WMH-related pathological processes constitute a more direct driver of cognitive decline, with GMV serving as the principal mediator independent of age-related effects. For instance, periventricular WMH induces frontal cortical thinning through combined vascular and neurodegenerative pathways,^52^ while thalamic and insular GMV loss further exacerbates cognitive deficits.^26^ These findings collectively establish GMV as the pivotal mediator translating WMH burden into cognitive impairment.

While our study focused on total WMH volume as a robust marker of global cerebrovascular burden, we acknowledge that spatial heterogeneity of WMH lesions, particularly their distribution along specific white matter tracts or periventricular zones, may confer differential effects on cortical integrity. Substantial evidence has demonstrated that strategic tract involvement can disproportionately accelerate regional cortical thinning in connected areas.^53,54^ The present volumetric approach, while methodologically consistent with established CSVD biomarkers,^1^ inherently cannot distinguish these location-specific effects. Future studies integrating tract-based WMH mapping could clarify how location-specific demyelination drives network vulnerability.

Furthermore, the relatively weak associations observed between age and both GMV and cognitive performance in this study appear to diverge from the broader literature on brain ageing. This discrepancy likely stems from the unique design of our study, which prioritized the isolation of WMH-specific effects by establishing a relatively young community-based cohort rigorously selected for the absence of other CSVD imaging markers. The resulting ‘purified’ sample, while minimizing confounding pathological influences, also restricted the range of age-related variance and general senescent pathologic burden. Consequently, within this specific population, WMH burden emerged as a more dominant driver of early structural and cognitive decline than chronological age. This finding underscores that in the early stages of CSVD, WMH may serve as a more sensitive and direct indicator of risk than age alone.

### Limitations

Several limitations should be acknowledged in this study. First, the small sample size reduces the reliability of the findings, requiring validation in larger, independent cohorts. Second, significant age differences exist between WMH patients and HCs. This reflects the epidemiological challenge of recruiting controls aged >50 years free of CSVD imaging markers. Although age was rigorously controlled as a covariate in all analyses, residual confounding effects cannot be entirely excluded. Third, while pseudo-time series data provided an innovative method for causal inference, CaSCN findings may not fully reflect the actual temporal progression of changes, emphasizing the need for longitudinal studies. Fourth, relying solely on WMH volume to measure disease severity may overlook underlying microstructural damage, emphasizing the need to incorporate diffusion tensor imaging for more detailed assessments. Finally, cognitive tools such as TMT-A and DST may have limited sensitivity in highly educated individuals, suggesting the need for additional cognitive assessments and the inclusion of vascular risk factors and other CSVD markers in future studies.

## Conclusion

In summary, this study elucidates a novel hierarchical pathway mechanism by which WMH drive structural brain alterations and cognitive decline. The right insula was identified as the central driver of early-stage grey matter atrophy. This region initiates degenerative pathways that impact motor learning, attention, executive function, and visual processing, ultimately leading to widespread brain atrophy. WMH primarily induces cognitive impairment via grey matter atrophy, particularly affecting processing speed and executive function. These findings not only underscore the critical role of grey matter volume changes in linking WMH burden to cognitive decline but also establish the right insula as the epicentre of structural damage.

## Supplementary Material

fcaf429_Supplementary_Data

## Data Availability

Requests to access the datasets will be reviewed by Ronghua Mu at Nanxishan Hospital of Guangxi Zhuang Autonomous Region and Xiqi Zhu at Affiliated Hospital of Youjiang Medical University for Nationalities to evaluate compliance with intellectual property protections and confidentiality agreements. Anonymized data may be shared with qualified researchers upon reasonable request for the sole purpose of replicating our findings, contingent upon adherence to applicable Chinese laws and ethical regulations. All code-related inquiries will undergo parallel evaluation by the respective authors at their affiliated institutions. No novel software, algorithms, in-house scripts, or custom programs were developed for this study.
