# Supplementary material for: White matter hyperintensities drive propagating grey matter atrophy in cerebral small vessel disease
Source: Brain Commun. 2025 Oct 30;7(6):fcaf429. doi: 10.1093/braincomms/fcaf429 (PMC12609173; doi:10.1093/braincomms/fcaf429)
Supplement: fcaf429_Supplementary_Data [file fcaf429_supplementary_data.docx]

**Supplementary Material**

**Supplementary Table 1. Sequences of multi-parametric MRI**

| Scan  sequences | Imaging plane | TR/TE, msec | FOV, mm | Slice  thickness, mm | Reconstruction matrix | Reconstruction voxel, mm^3^ |
| --- | --- | --- | --- | --- | --- | --- |
| 3D T1 SE | Sagittal | 6.4/3.0 | 240 × 240 × 160 | 1.0 | 240 × 240 | 1.0 × 1.0 × 1.0 |
| 3D T2 SE | Sagittal | 2,500/232 | 240 × 240 × 160 | 1.0 | 240 × 240 | 1.0 × 1.0 × 1.0 |
| 3D FLAIR | Sagittal | 4,800/244 | 240 × 240 × 160 | 1.0 | 240 × 240 | 1.0 × 1.0 × 1.0 |
| SWI | Axial | 29/7.2 | 230 × 230 × 130 | 2.0 | 768 × 768 | 0.3 × 0.3 × 1.0 |
| DWI | Axial | 2462/63 | 230 × 230 × 145 | 5.0 | 192×192 | 1.2×1.2×5.0 |

**Supplementary Table 2. Demographic and clinical characteristics of WMH subgroups**

| Characteristic | Stage I  (n=55) | Stage II  (n=43) | Stage III  (n=50) | Stage Ⅳ (n=37) | HC  (n=40) | Comparisons (*p*-value) | | | |
| --- | --- | --- | --- | --- | --- | --- | --- | --- | --- |
|  |  |  |  |  |  | Stage Ⅰ  vs HC | Stage ⅠI  vs HC | Stage ⅡI  vs HC | Stage Ⅳ  vs HC |
| Age (years) | 56.5 ± 6.4 | 57.4 ± 6.7 | 58.2 ± 6.5 | 59.1 ± 6.3 | 45.5 ± 7.8 | **<0.001^a^** | **<0.001^a^** | **<0.001^a^** | **<0.001^a^** |
| Gender (male/female) | 25/30 | 17/26 | 24/26 | 20/17 | 21/19 | 0.479^b^ | 0.236^b^ | 0.671^b^ | 0.891^b^ |
| Educational level (years) | 12.4 ± 3.2 | 10.9 ± 3.3 | 9.9 ± 3.5 | 9.9 ± 4.1 | 11.5 ± 5.4 | 0.991^a^ | 0.953^a^ | 0.673^a^ | 0.747^a^ |
| MoCA (0–30) | 27.26 ± 2.05 | 27.19 ± 2.04 | 27.12 ± 1.48 | 25.43 ± 2.04 | 27.50 ± 1.43 | 1.000^a^ | 1.000**^a^** | 1.000**^a^** | **0.006^a^** |
| MMSE (0–30) | 27.00 ± 1.88 | 28.07 ± 1.62 | 27.18 ± 1.71 | 24.81 ± 1.41 | 28.13 ± 1.07 | 0.653**^a^** | 0.933**^a^** | 0.928**^a^** | **<0.001^a^** |
| DST (correct matches/2 minutes) | 50.38 ± 4.71 | 43.74 ± 6.70 | 37.08 ± 9.12 | 30.65 ± 5.41 | 54.48 ± 15.12 | 0.226**^a^** | **<0.001^a^** | **<0.0001^a^** | **<0.001^a^** |
| TMT-A (seconds) | 36.64 ± 10.03 | 52.59 ± 13.99 | 55.69 ± 11.94 | 62.29 ± 25.64 | 41.42 ± 12.33 | 0.386^a^ | **0.002^a^** | **0.001^a^** | **<0.001^a^** |
| WMH volume (mm³) | 1.48 ± 0.95 | 4.13 ± 0.88 | 10.62 ± 2.33 | 19.13 ± 5.43 | 0 | NA | NA | NA | NA |
| GMV (mm³) | 561.60 ± 25.77 | 552.73 ± 14.87 | 543.63 ± 12.25 | 532.80 ± 40.11 | 592.64 ± 46.92 | **<0.001^a^** | **<0.001^a^** | **<0.001^a^** | **<0.001^a^** |
| TIV (mm³) | 1446.50 ± 116.56 | 1428.39 ± 96.44 | 1411.82 ± 113.01 | 1405.63 ± 113.03 | 1505.07 ± 147.42 | 0.177 | **0.034^a^** | **0.002^a^** | **0.003^a^** |

Abbreviations: DST, digit symbol substitution test; GMV, gray matter volume; HC, healthy control; MMSE, Mini-Mental State Examination; MoCA, Montreal Cognitive Assessment; TIV, total intracranial volume; TMT-A, trail making test-A; WMH, white matter hyperintensity.

Data are presented as median [25th percentile, 75th percentile] for WMH volume and as mean ± standard deviation for all other continuous variables, with gender specified as male/female counts.

Significant differences are highlighted in bold.

^a^ One-Way ANOVA.

^b^ Chi-square *t*-test.

**Supplementary Table 3. Demographic and Clinical Characteristics of Subgroups Based on the WMH Fazekas grading**

| Characteristic | Stage I  (n=88) | Stage II  (n=52) | Stage III  (n=45) | HC  (n=40) | Comparisons (*p*-value) | | |
| --- | --- | --- | --- | --- | --- | --- | --- |
|  |  |  |  |  | Stage Ⅰ  *vs* HC | Stage Ⅱ  *vs* HC | Stage III  *vs* HC |
| Age (years) | 56.9 ± 6.6 | 57.9 ± 6.7 | 59.0 ± 6.0 | 45.5 ± 7.8 | **<0.001^a^** | **<0.001^a^** | **<0.001^a^** |
| Gender (male/female) | 38/50 | 23/29 | 25/20 | 21/19 | 0.327^b^ | 0.431^b^ | 0.671^b^ |
| Educational level (years) | 11.7 ± 3.3 | 9.7 ± 3.6 | 10.1 ± 3.8 | 11.5 ± 5.4 | 0.999^a^ | 0.189^a^ | 0.530^a^ |
| MoCA (0–30) | 27.58 ± 1.30 | 27.50 ± 1.36 | 26.82 ± 0.98 | 27.50 ± 1.43 | 1.000^a^ | 1.000**^a^** | 0.096**^a^** |
| MMSE (0–30) | 28.16 ± 1.12 | 28.06 ± 1.11 | 27.36 ± 0.71 | 28.13 ± 1.07 | 1.000^a^ | 1.000**^a^** | **0.005^a^** |
| DST (correct matches/2 minutes) | 48.09 ± 5.91 | 38.02 ± 9.70 | 38.10 ± 5.96 | 54.48 ± 15.12 | **0.002^a^** | **<0.001^a^** | **<0.0001^a^** |
| TMT-A (seconds) | 42.89 ± 13.61 | 55.76 ± 13.53 | 59.81 ± 24.13 | 41.42 ± 12.33 | 0.999^a^ | **<0.001^a^** | **<0.001^a^** |
| WMH volume (mm³) | 2.34 ± 1.38 | 8.99 ± 2.28 | 18.31 ± 5.30 | 0 | NA | NA | NA |
| GMV (mm³) | 558.18 ± 20.98 | 544.93 ± 11.91 | 535.43 ± 37.08 | 592.64 ± 46.92 | **<0.001^a^** | **<0.001^a^** | **<0.001^a^** |
| TIV (mm³) | 1440.01 ± 110.07 | 1408.47 ± 102.57 | 1413.69 ± 121.71 | 1505.07 ± 147.42 | **0.025^a^** | **0.001^a^** | **0.003^a^** |

Abbreviations: DST, digit symbol substitution test; GMV, gray matter volume; HC, healthy control; MMSE, Mini-Mental State Examination; MoCA, Montreal Cognitive Assessment; TIV, total intracranial volume; TMT-A, trail making test-A; WMH, white matter hyperintensity.

Data are presented as median [25th percentile, 75th percentile] for WMH volume and as mean ± standard deviation for all other continuous variables, with gender specified as male/female counts.

Significant differences are highlighted in bold.

^a^ One-Way ANOVA.

^b^ Chi-square *t*-test.

**Supplementary Table 4. Results of group comparison of GMV between patients with WMH and HCs**

| **Brain Regions** | **MNI coordinates (x,y,z)** | | | ***T*-value** | **Number of voxels** |
| --- | --- | --- | --- | --- | --- |
| Insula, right | 45 | 18 | -4.5 | 7.153 | 14395 |
| Inferior temporal gyrus, right | 64.5 | -24 | -21 | 5.439 | 263 |
| Middle temporal gyrus, left | -66 | -40.5 | 4.5 | 5.807 | 699 |
| Fusiform gyrus, left | -24 | -42 | -13.5 | 5.540 | 138 |
| Inferior occipital gyrus, right | 45 | -75 | -16.5 | 5.661 | 366 |
| Fusiform gyrus, right | 24 | -39 | -12 | 6.138 | 514 |
| Superior frontal gyrus, medial orbital, right | 45 | 54 | -15 | 5.083 | 93 |
| Inferior occipital gyrus, left | -48 | -73.5 | -6 | 5.483 | 319 |
| Superior frontal gyrus, medial, right | 3 | 67.5 | 3 | 5.690 | 1175 |
| Middle occipital gyrus, right | 30 | -99 | 0 | 5.032 | 87 |
| Lingual gyrus, right | 13.5 | -34.5 | -1.5 | 5.033 | 51 |
| Inferior frontal gyrus, triangular part, left | -51 | 46.5 | 1.5 | 5.309 | 79 |
| Insula, left | -33 | -19.5 | 6 | 5.919 | 1572 |
| Middle temporal gyrus, right | 64.5 | -49.5 | 13.5 | 5.300 | 128 |
| Median cingulate and paracingulate gyri, left | -7.5 | -15 | 34.5 | 6.082 | 1554 |
| Supplementary motor area, left | 0 | 10.5 | 54 | 5.823 | 1301 |
| Superior frontal gyrus, medial, left | -7.5 | 48 | 30 | 5.367 | 337 |
| Middle frontal gyrus, right | 33 | 51 | 27 | 5.280 | 142 |
| Cuneus, left | -7.5 | -67.5 | 28.5 | 5.321 | 82 |
| Angular gyrus, left | -52.5 | -64.5 | 31.5 | 5.032 | 74 |
| Precuneus, right | 7.5 | -66 | 31.5 | 5.112 | 98 |
| Precental gyrus, right | 37.5 | -22.5 | 64.5 | 6.065 | 689 |
| Inferior parietal, but supramarginal and angular gyri, left | -46.5 | -43.5 | 48 | 5.168 | 83 |
| Precuneus, left | -1.5 | -67.5 | 58.5 | 5.074 | 101 |
| Superior frontal gyrus, dorsolateral, right | 28.5 | 24 | 52.5 | 5.383 | 75 |
| Postcentral gyrus, left | -21 | -25.5 | 79.5 | 5.167 | 195 |

Abbreviations: GMV, gray matter volume; HCs, healthy controls; MNI, Montreal Neurologic Institute; WMH, white matter hyperintensities.

**Supplementary Table 5. Results of group comparison of GMV between patients with WMH and HCs grouped by WMH volume**

| **Brain Regions** | **MNI coordinates (x,y,z)** | | | ***T*-value** | **Number of voxels** |
| --- | --- | --- | --- | --- | --- |
| ***Stage I*** | | | | | |
| Insula, right | 46.5 | 15 | -6 | 5.829 | 106 |
| ***Stage II*** | | | | | |
| Insula, right | 46.5 | 16.5 | -4.5 | 6.074 | 320 |
| Gyrus rectus, left | -9 | 27 | -19.5 | 5.431 | 222 |
| Insula, left | -43.5 | 16.5 | -3 | 5.901 | 262 |
| Fusiform gyrus, right | 24 | -40.5 | -13.5 | 5.755 | 202 |
| Middle frontal gyrus, right | 54 | 46.5 | 9 | 5.514 | 138 |
| Median cingulate and paracingulate gyri, left | 0 | -27 | 45 | 6.206 | 764 |
| Middle frontal gyrus, left | 28.5 | 16.5 | 52.5 | 5.720 | 105 |
| ***Stage III*** | | | | | |
| Insula, right | 43.5 | 19.5 | -3 | 6.802 | 1628 |
| Temporal pole, middle temporal gyrus, left | -42 | 0 | 4.5 | 7.934 | 5699 |
| Middle temporal gyrus, left | -66 | -37.5 | -13.5 | 6.294 | 1277 |
| Parahippocampal gyrus, right | 25.5 | -39 | -6 | 5.631 | 185 |
| Inferior occipital gyrus, left | -48 | -72 | -7.5 | 6.069 | 413 |
| Fusiform gyrus, left | -24 | -39 | -12 | 5.566 | 149 |
| Superior frontal gyrus, medial orbital, right | 43.5 | 51 | -16.5 | 5.899 | 247 |
| Superior temporal gyrus, right | 63 | -46.5 | 12 | 5.887 | 213 |
| Precuneus, left | -1.5 | -60 | 15 | 6.035 | 135 |
| Inferior frontal gyrus, triangular part, left | -48 | 19.5 | 27 | 6.790 | 441 |
| Median cingulate and paracingulate gyri, left | -7.5 | -15 | 34.5 | 6.233 | 439 |
| Supplementary motor area, right | 1.5 | 13.5 | 57 | 6.288 | 962 |
| Middle frontal gyrus, left | -25.5 | 22.5 | 51 | 6.194 | 673 |
| Precentral, right | 45 | -18 | 48 | 5.895 | 369 |
| Precentral, left | -27 | -28.5 | 64.5 | 5.989 | 408 |
| ***Stage Ⅳ*** | | | | | |
| Insula, right | 57 | 22.5 | 3 | 6.111 | 1711 |
| Inferior frontal gyrus, orbital part, left | -45 | 15 | -3 | 7.412 | 8785 |
| Middle temporal gyrus, right | 57 | -22.5 | -15 | 6.417 | 498 |
| Middle temporal gyrus, left | -60 | -36 | -7.5 | 6.457 | 1029 |
| Lingual gyrus, right | 9 | -79.5 | -1.5 | 6.170 | 1400 |
| Middle frontal gyrus, orbital part, left | -3 | 52.5 | -3 | 5.325 | 123 |
| Lingual, left | -10.5 | -79.5 | 1.5 | 6.327 | 497 |
| Middle occipital gyrus, left | -22.5 | -99 | 0 | 5.720 | 109 |
| Precuneus, right | 16.5 | -40.5 | 1.5 | 5.312 | 162 |
| Rolandic operculum, right | 49.5 | -12 | 9 | 5.522 | 162 |
| Superior frontal gyrus, medial, right | 6 | 66 | 6 | 6.198 | 524 |
| Superior frontal gyrus, dorsolateral, right | 18 | 55.5 | 9 | 5.718 | 153 |
| Supramarginal gyrus, left | -61.5 | -22.5 | 15 | 6.417 | 185 |
| Precuneus, left | -1.5 | -61.5 | 15 | 5.495 | 182 |
| Superior frontal gyrus, medial, left | 1.5 | 57 | 30 | 5.626 | 210 |
| Median cingulate and paracingulate gyri, left | -9 | -13.5 | 34.5 | 7.324 | 1825 |
| Precental gyrus, right | 46.5 | -10.5 | 51 | 6.119 | 764 |
| Supplementary motor area, left | 1.5 | 31.5 | 61.5 | 5.897 | 897 |

All patients were categorized into four subgroups based on WMH volume (with WMH volume fluctuation thresholds of 3.21, 7.78, and 14.06). Region I-IV indicates that the region was detected at stages I-IV.

Abbreviations: GMV, gray matter volume; HCs, healthy controls; MNI, Montreal Neurologic Institute; WMH, white matter hyperintensities.

**Supplementary Table 6. Results of group comparison of GMV between patients with WMH and HCs grouped by Fazekas grading**

| **Brain Regions** | **MNI coordinates (x,y,z)** | | | ***T*-value** | **Number of voxels** |
| --- | --- | --- | --- | --- | --- |
| ***Stage I*** | | | | | |
| Insula, right | 43.5 | 19.5 | -3 | 6.165 | 433 |
| ***Stage II*** | | | | | |
| Insula, right | 42 | 7.5 | 3 | 6.178 | 1011 |
| Inferior frontal gyrus, orbital part, right | 45 | 19.5 | -15 | 5.578 | 140 |
| Inferior temporal gyrus, right | 57 | -22.5 | -15 | 6.060 | 253 |
| Parahippocampal gyrus, right | 15 | 3 | -18 | 6.195 | 778 |
| Middle temporal gyrus, left | -61.5 | -13.5 | -10.5 | 5.723 | 176 |
| Inferior frontal gyrus, triangular part, left | -49.5 | 19.5 | 30 | 5.607 | 122 |
| Insula, left | -45 | 15 | -1.5 | 7.343 | 2290 |
| Middle occipital gyrus, left | -23 | -100.5 | 0 | 5.590 | 105 |
| Cuneus, right | 18 | -58.5 | 19.5 | 5.214 | 234 |
| Median cingulate and paracingulate gyri, left | 0 | -28.5 | 45 | 5.812 | 153 |
| ***Stage III*** | | | | | |
| Insula, right | 40.5 | 4.5 | 1.5 | 7.150 | 2791 |
| Insula, left | -43.5 | -3 | 0 | 7.425 | 6521 |
| Median cingulate and paracingulate gyri, left | -7.5 | -18 | 34.5 | 6.390 | 725 |
| Middle frontal gyrus, left | -25.5 | 21 | 52.5 | 6.365 | 742 |
| Middle temporal gyrus, right | 57 | -22.5 | -15 | 5.350 | 101 |
| Middle temporal gyrus, left | -66 | -40.5 | 4.5 | 6.257 | 1079 |
| Inferior occipital gyrus, left | -48 | -72 | -6 | 6.253 | 402 |
| Fusiform gyrus, left | -24 | -39 | -16.5 | 5.454 | 138 |
| Inferior frontal gyrus, triangular part, left | -48 | -21 | 27 | 7.056 | 580 |
| Supramarginal gyrus, left | -58.5 | -39 | 24 | 5.489 | 103 |
| Supplementary motor area, right | 1.5 | 13.5 | 55.5 | 5.864 | 733 |
| Precentral, right | 45 | -10.5 | 49.5 | 6.380 | 552 |
| Precentral, left | -28.5 | -28.5 | 63 | 5.808 | 343 |

All patients were categorized into three subgroups based on Fazekas scale. Region I-III indicates that the region was detected at stages I-III.

Abbreviations: GMV, gray matter volume; HCs, healthy controls; MNI, Montreal Neurologic Institute; WMH, white matter hyperintensities.

**Supplementary Table 7. Mediation Analysis Results**

| Outcome | Path | Coefficient (β) | T value | 95% CI | *p*-value | % Effect |
| --- | --- | --- | --- | --- | --- | --- |
| DST | WMH → GMV | -0.96 | -42.44 | -1.00, -0.91 | **<0.001** | -99.1% |
|  | GMV → DST | -0.62 | -12.67 | -0.71, -0.52 | **<0.001** | -63.9% |
|  | WMH → DST (direct) | 0.38 | 7.83 | 0.28, 0.47 | **<0.001** | 39.0% |
|  | WMH → GMV → DST (indirect) | 0.59 | 12.14 | 0.49, 0.68 | **<0.001** | 61.0% |
|  | Total effect | 0.96 | 47.70 | 0.92, 1.00 | **<0.001** | 100% |
| 1/TMT-A | WMH → GMV | -0.95 | -42.44 | -1.00, -0.91 | **<0.001** | -99.0% |
|  | GMV → 1/TMT-A | -0.67 | -15.88 | 0.76, -0.59 | **<0.001** | -69.8% |
|  | WMH → 1/TMT-A (direct) | 0.32 | 7.16 | 0.24, 0.41 | **<0.001** | 33.4% |
|  | WMH → GMV → 1/TMT-A (indirect) | 0.64 | 14.87 | 0.56, 0.73 | **<0.001** | 66.6% |
|  | Total effect | 0.96 | 47.49 | 0.93, 1.00 | **<0.001** | 100% |

Abbreviations: CI, confidence interval; DST, digit symbol substitution test; GMV, gray matter volume; TMT-A, trail making test-A; WMH, white matter hyperintensity.


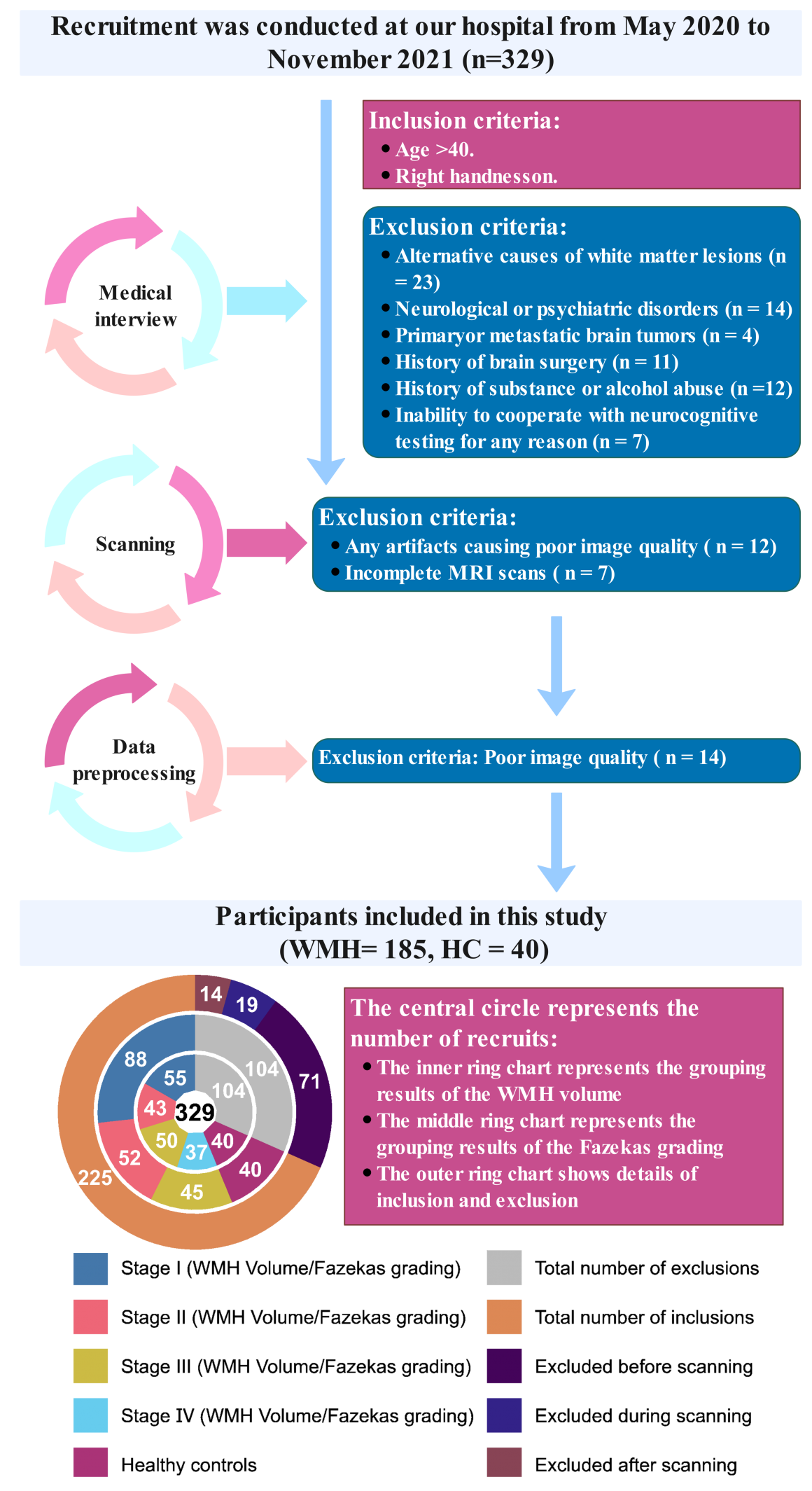


**Supplementary Figure 1. Procedure of inclusion and exclusion of the participants.** Participant Recruitment Flowchart and Grouping Criteria This figure integrates two components: a recruitment flowchart and a multi-ring diagram illustrating participant grouping. The recruitment pipeline (right panel) summarizes enrollment conducted at our hospital from May 2020 to November 2021 (total screened: *N* = 329). Participants were sequentially evaluated through three stages with corresponding exclusion criteria: (1) medical interview (exclusions: alternative white matter lesion causes [*N* = 23], neurological/psychiatric disorders [*N* = 14], intracranial tumors [*N* = 9], substance/alcohol abuse [*N* = 12], inability to cooperate [*N* = 7]); (2) MRI scanning (exclusions: image artifacts [*N* = 12], incomplete scans [*N* = 7]); and (3) data preprocessing (exclusion: poor image quality [*N* = 14]). The final cohort included 185 WMH patients and 40 HCs. The multi-ring grouping diagram (left panel) uses concentric circles to visualize WMH patient characteristics: the central circle denotes the total number of included WMH patients (*N* = 185); the inner ring represents staging by local polynomial regression (Stage I–IV); the middle ring shows modified Fazekas grading (Stage I–III); and the outer ring details inclusion/exclusion criteria distribution. Color coding corresponds to grouping categories as indicated in the figure inset.

Abbreviations: HCs, healthy controls; WMH, white matter hyperintensities.

**
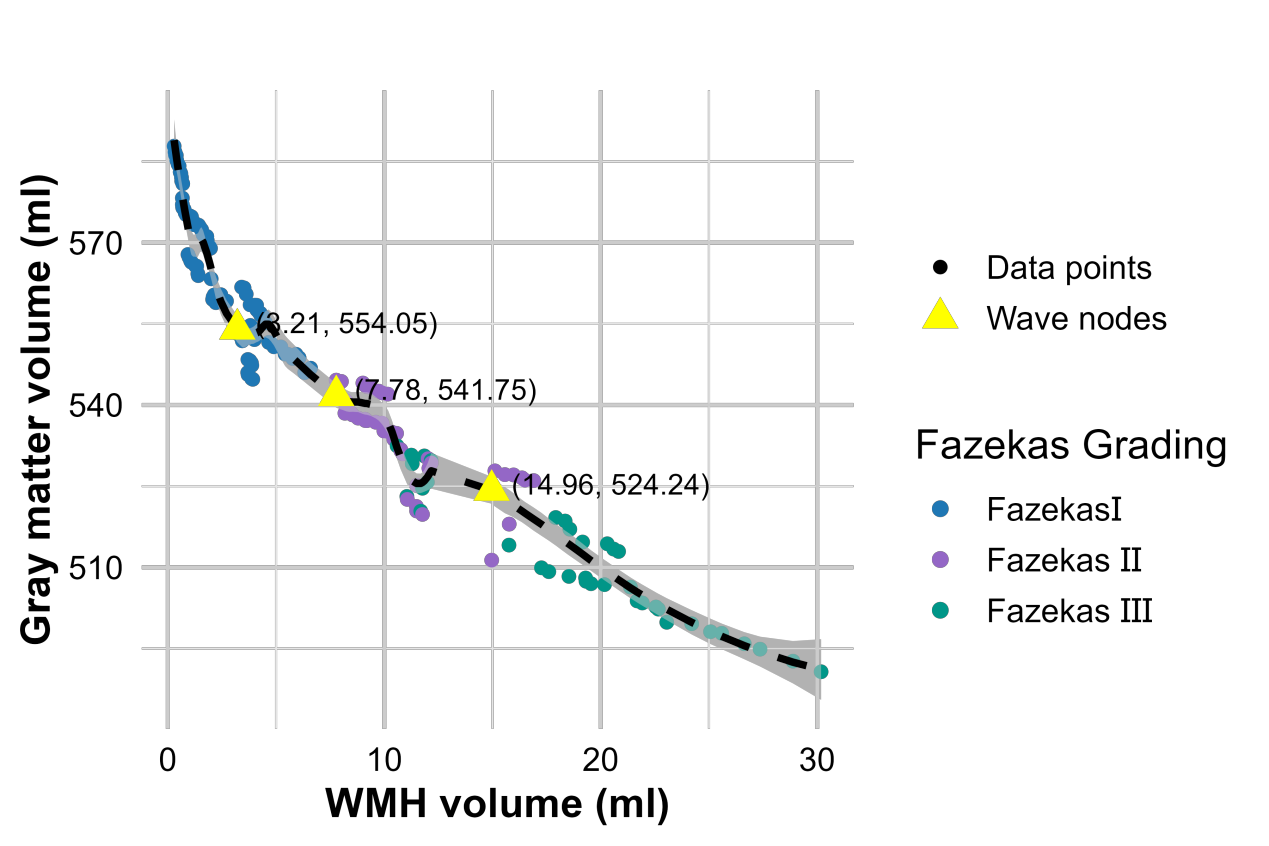
**

**Supplementary Figure 2. Analysis of GMV relative to WMH volume using locally weighted polynomial regression.**

This figure illustrates the relationship between mean GMV and WMH volume, analyzed using locally weighted polynomial regression (*N* = 185). The analysis reveals three inflection points in GMV trends (F = 442.280, df1 = 15.792, df2 = 168.208, *p* < 0.001, R^2^ = 0.97 ): The first fluctuation occurs at a WMH volume of 3.21 ml. The second fluctuation occurs at a WMH volume of 7.78 ml. The third fluctuation occurs at a WMH volume of 14.96 ml.

Each colored point represents data from individual participants, with azure, amethyst, and teal points corresponding to different Fazekas grading. The yellow triangles mark the identified inflection points, highlighting key changes in GMV trends as WMH volume increases.

Abbreviations: HCs, healthy controls; GMV, gray matter volume; WMH, white matter hyperintensities.


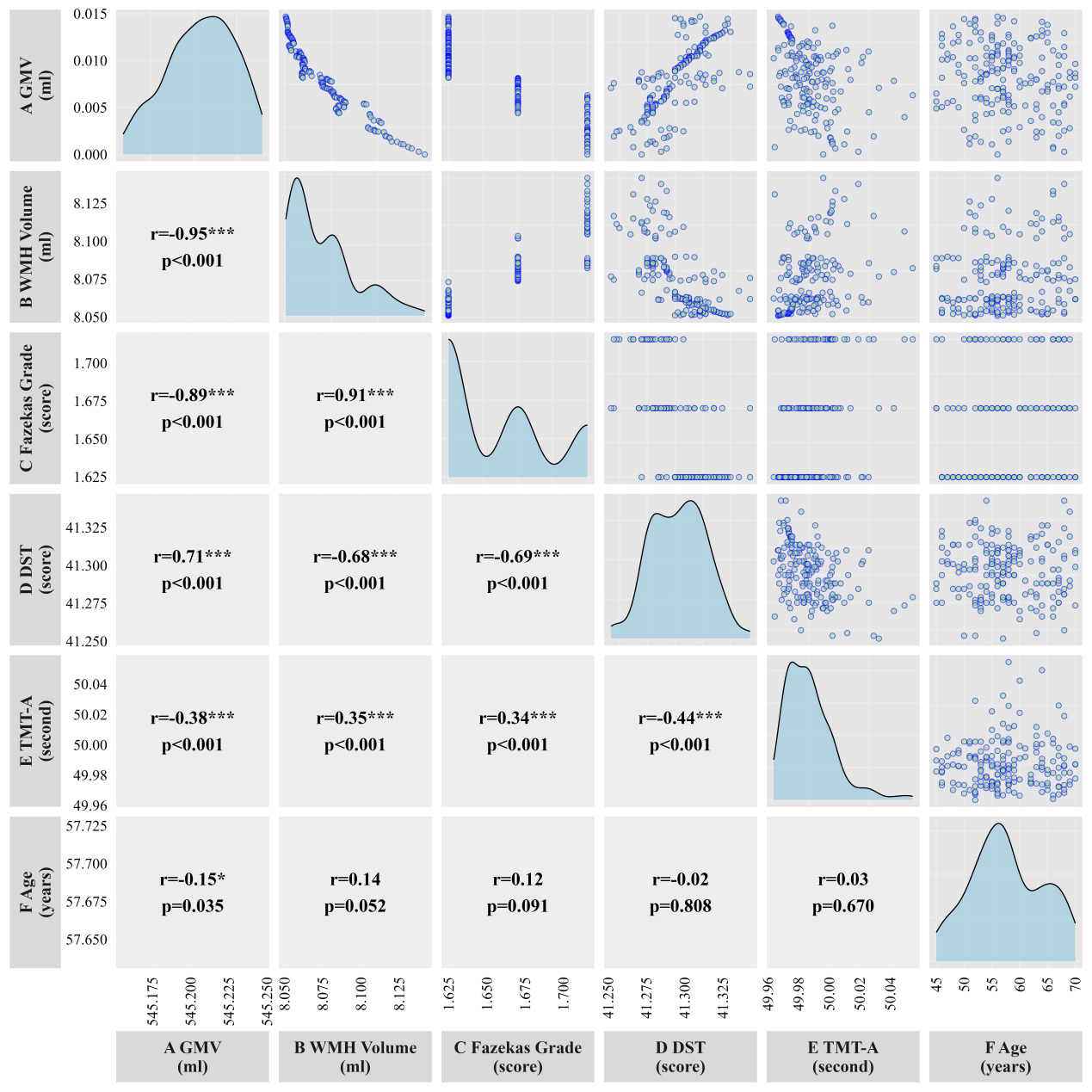


**Supplementary Figure 3. Scatterplot matrix of brain volumetric, cognitive, and age variables.**

The scatterplot matrix presents pairwise comparisons between the following variables: GMV, WMH volume, Fazekas grade, DST, TMT-A, and Age (*N =* 185), where each blue data point represents an individual patient with WMH. A. GMV: Refers to the volume of grey matter in the brain. B. WMH Volume: Indicates the volume of white matter hyperintensities. C. Fazekas grade: A grading system used to assess the severity of WMH on MRI, ranging from 0 (no lesions) to 3 (severe lesions). D. DST: Measures cognitive processing speed and executive function. E. TMT-A: Assesses attention and visual-motor tracking abilities. F. Age: Represents the chronological age (in years) of patients with white matter hyperintensity at the time of data collection.

Pairwise correlations were evaluated using Spearman correlation analysis, with statistical significance defined as *p* < 0.05. The diagonal panels feature kernel density plots illustrating the univariate distribution of each variable. The upper triangular panels display scatterplots capturing bivariate relationships between variable pairs, while the lower triangular panels present Spearman's rank correlation coefficients with corresponding significance asterisks.

Abbreviations: DST, Digit Symbol Test; GMV, gray matter volume; TMT-A, Trail making test-A; WMH, white matter hyperintensities.

*, < 0.05; ***, < 0.001.


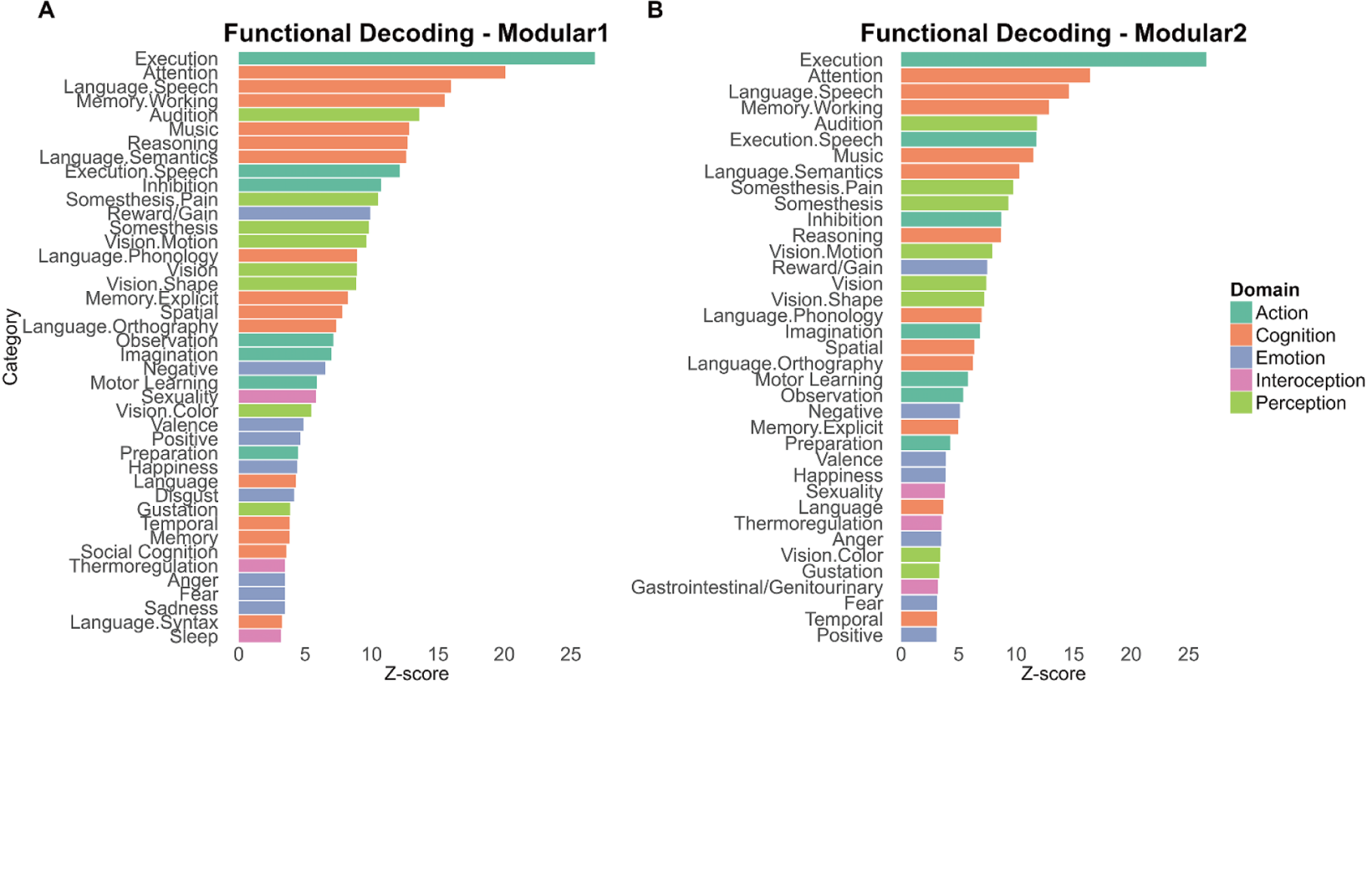
**Supplementary Figure 4. Functional decoding.**

All analyses were performed in patients with White matter hyperintensities (*N* = 185). **A** and **B**. Functional decoding of the identified brain modules. Bar plots display the functional decoding results, illustrating the primary cognitive functions associated with each module. Significant associations (Z-scores > 3.0, *p* < 0.05), assessed using a binomial test with false discovery rate correction for multiple comparisons, indicate that both Module 1 and Module 2 are strongly linked to executive function and attention.
